# Supplementary material for: Mortality in children under 5 years of age with congenital syphilis in Brazil: A nationwide cohort study
Source: PLoS Med. 2023 Apr 7;20(4):e1004209. doi: 10.1371/journal.pmed.1004209 (PMC10081765; doi:10.1371/journal.pmed.1004209)
Supplement: S1 Fig — (PDF) [file pmed.1004209.s001.pdf]

Figure S1: Flow diagram

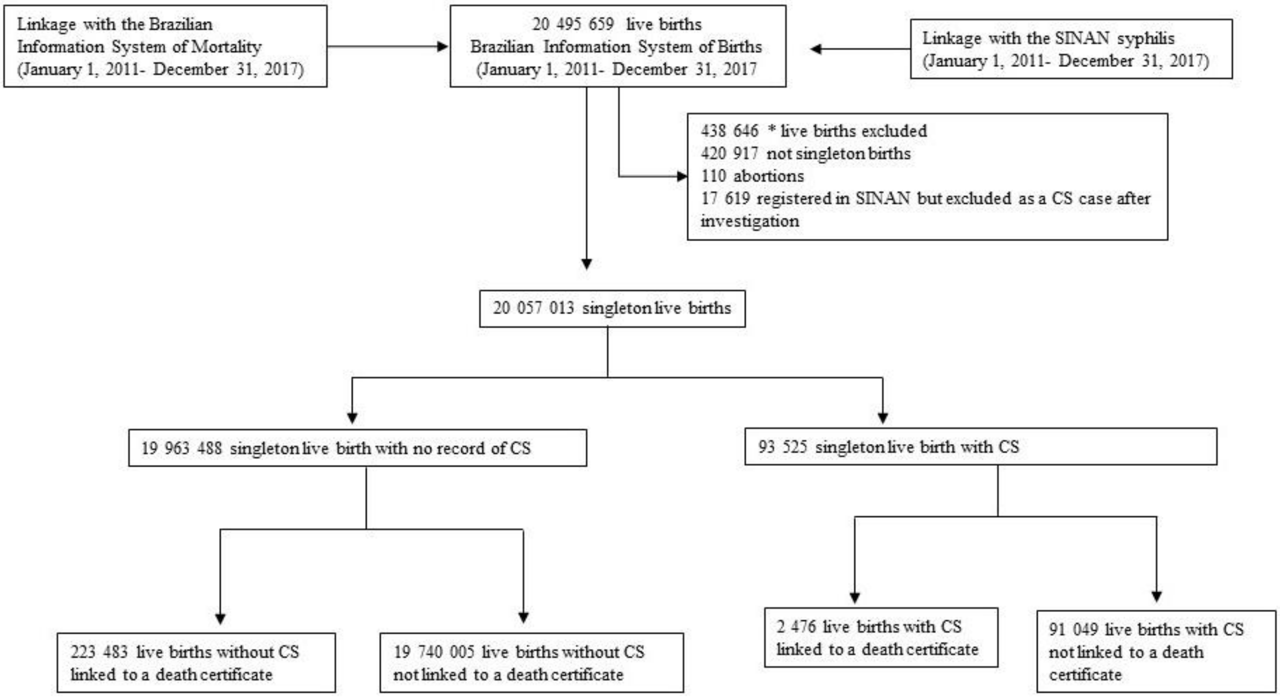

CS: Congenital syphilis

SINAN: Information System for Notifiable Disease
