# Supplementary material for: Mortality in children under 5 years of age with congenital syphilis in Brazil: A nationwide cohort study
Source: PLoS Med. 2023 Apr 7;20(4):e1004209. doi: 10.1371/journal.pmed.1004209 (PMC10081765; doi:10.1371/journal.pmed.1004209)
Supplement: S2 Fig — (PDF) [file pmed.1004209.s002.pdf]

Figure S2: Proportional mortality ratio comparing Congenital Syphilis status

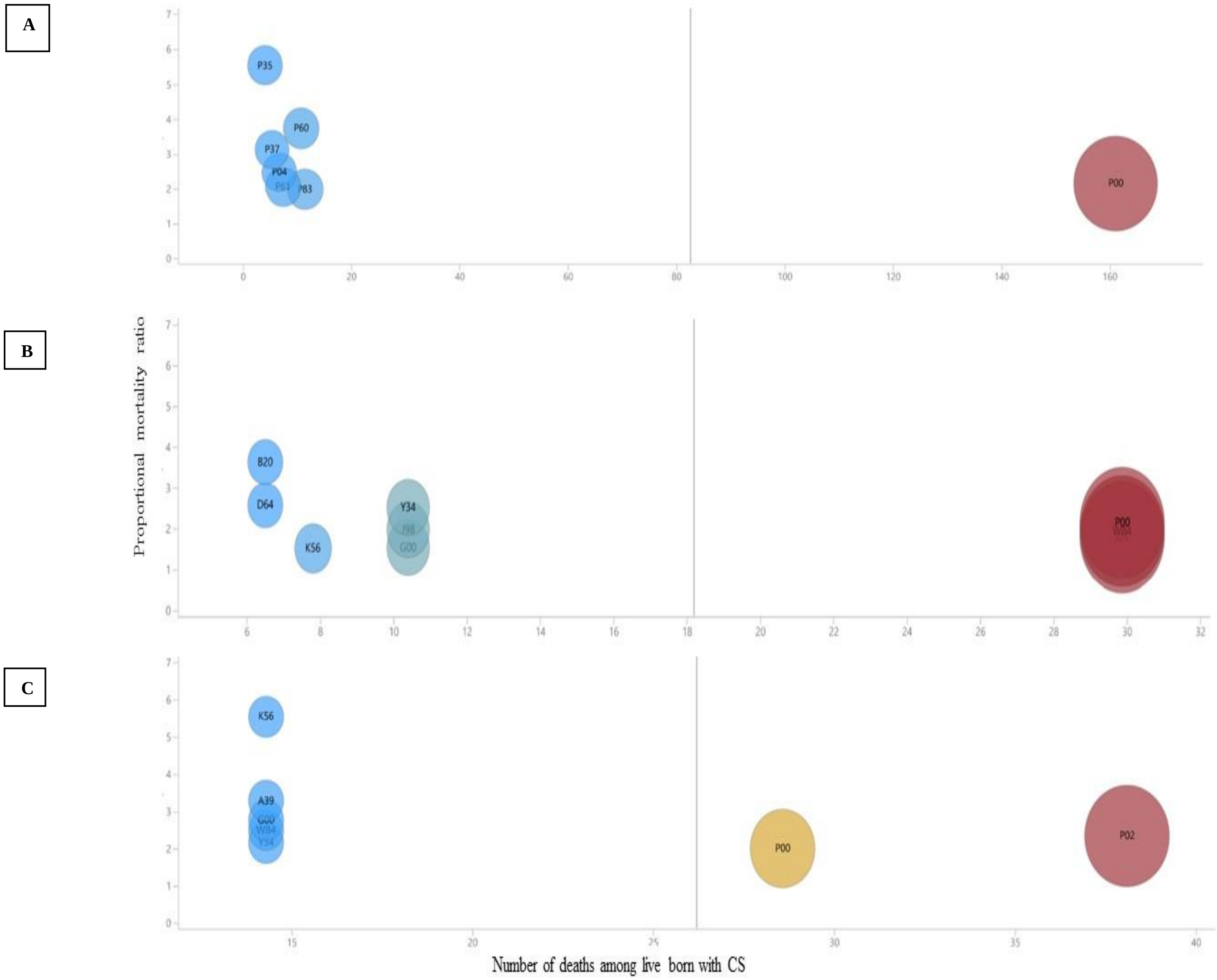

A: Neonatal period – P00 Newborn affected by maternal conditions that may be unrelated to present pregnancy P04 Newborn affected by noxious substances transmitted via placenta or breast milk P35 Congenital viral diseases P37 Other congenital infectious and parasitic diseases P60 Disseminated intravascular coagulation of newborn P61 Other perinatal hematological disorders P83 Other conditions of integument specific to newborn. **B: Post neonatal period**- B20 Human immunodeficiency virus [HIV] disease D64 Other anaemias G00 Bacterial meningitis, not elsewhere classified J21 Acute bronchiolitis J98 Other respiratory disorders K56 Paralytic ileus and intestinal obstruction without hernia P00 Newborn affected by maternal conditions that may be unrelated to present pregnancy W84 Unspecified threat to breathing Y34 Unspecified event, undetermined intent **C: 1-4 years** – A39 Meningococcal infection G00 Bacterial meningitis, not elsewhere classified K56 Paralytic ileus and intestinal obstruction without hernia P00 Newborn affected by maternal conditions that may be unrelated to present pregnancy P02 Newborn affected by complications of placenta, cord and membranes W84 Unspecified threat to breathing Y34 Unspecified event, undetermined intent
