# Supplementary material for: Mortality in children under 5 years of age with congenital syphilis in Brazil: A nationwide cohort study
Source: PLoS Med. 2023 Apr 7;20(4):e1004209. doi: 10.1371/journal.pmed.1004209 (PMC10081765; doi:10.1371/journal.pmed.1004209)
Supplement: S2 Table — (DOCX) [file pmed.1004209.s004.docx]

**Table S2: Baseline characteristics of deaths**

|  | **Deaths among those without CS**  **n (%)** | **Deaths among CS live births with SINAN register**  **n (%)** | **Death by CS without SINAN registry**  **n (%)** |
| --- | --- | --- | --- |
| **Maternal age (years)** |  |  |  |
| < 20 | 52,319 (23.49) | 751 (30.33) | 263 (36.03) |
| 20-34 | 139,198 (62.49) | 1,525 (61.59) | 419 (57.40) |
| 35+ | 31,224 (14.02) | 200 (8.08) | 48 (6.58) |
| Missing | 12 | - | - |
| **Marital status** |  |  |  |
| Single | 107,538 (48.99) | 1,800 (73.62) | 554 (77.37) |
| Widow | 427 (0.19) | 8 (0.33) | 3 (0.42) |
| Divorced | 2,204 (1.00) | 18 (0.74) | 7 (0.98) |
| Married/union | 109,323 (49.81) | 619 (25.32) | 152 (21.23) |
| Missing | 3,261 | 31 | 14 |
| **Maternal education** |  |  |  |
| none | 3,248 (1.29) | 35 (1.44) | 3 (0.42) |
| 1 - 3 years | 11,130 (5.10) | 163 (6.71) | 54 (7.52) |
| 4 - 7 years | 55,621 (25.50) | 1,020 (41.96) | 287 (39.97) |
| 8 - 12 years | 122,140 (56.00) | 1,173 (48.25) | 358 (49.86) |
| 12 + years | 25,979 (11.91) | 40 (1.65) | 16 (2.23) |
| Missing | 4,635 | 45 | 12 |
| **Maternal ethnicity** |  |  |  |
| White | 60,426 (30.44) | 462 (20.13) | 106 (15.61) |
| Black | 13,370 (6.73) | 265 (1.93) | 72 (10.60) |
| Asian | 631 (0.32) | 5 (0.22) | - |
| Mixed Brown | 120,491 (60.69) | 1,555 (67.76) | 493 (72.61) |
| Indigenous | 3,620 (1.82) | 8 (0.35) | 8 (1.18) |
| Missing | 24,215 | 181 | 51 |
| **Previous Stillbirth** |  |  |  |
| Yes | 46,140 (22.87) | 1,710 (74.74) | 169 (25.57) |
| No | 155,577 (77.13) | 578 (25.26) | 492 (74.43) |
| Missing | 21,036 | 188 | 69 |
| **Number of prenatal appointments** |  |  |  |
| None | 16,415 (7.38) | 406 (16.42) | 115 (15.75) |
| 0-3 appointments | 42,562 (19.13) | 767 (31.01) | 240 (32.88) |
| 4 - 6 appointments | 77,338 (34.76) | 724 (29.28) | 210 (28.77) |
| 7+ appointments | 86,157 (38.73) | 576 (23.29) | 165 (22.60) |
| Missing | 281 | 3 | - |
| **Mode of delivery** |  |  |  |
| Vaginal | 113,962 (51.28) | 1,551 (62.77) | 465 (63.79) |
| C-section | 108,275 (48.72) | 920 (37.23) | 264 (36.21) |
| Missing | 516 | 5 | 1 |
| **Year of birth** |  |  |  |
| 2011 | 31,953 (14.34) | 217 (8.76) | 41 (5.62) |
| 2012 | 32,011 (14.37) | 272 (10.99) | 66 (9.04) |
| 2013 | 31,824 (14.29) | 302 (12.20) | 86 (11.78) |
| 2014 | 31,936 (14.34) | 342 (13.81) | 113 (15.48) |
| 2015 | 32,100 (14.41) | 423 (17.08) | 138 (18.90) |
| 2016 | 32,357 (14.53) | 449 (18.13) | 145 (19.86) |
| 2017 | 30,572 (13.72) | 471 (19.02) | 141 (19.32) |
| Missing | - | - | - |
| **Birth Region** |  |  |  |
| N | 27,941 (12.55) | 210 (8.48) | 87 (11.92) |
| NE | 68,976 (30.97) | 754 (30.45) | 203 (27.81) |
| SE | 82,752 (37.16) | 1,065 (43.01) | 347 (47.53) |
| S | 25,415 (11.41) | 280 (11.31) | 52 (7.12) |
| MW | 17,637 (7.92) | 167 (6.74) | 41 (5.62) |
| Missing | 32 | - | - |
| **Sex of the newborn** |  |  |  |
| Female | 98,959 (44.54) | 1,154 (46.72) | 325 (44.58) |
| Male | 123,206 (55.46) | 1,316 (53.28) | 404 (55.42) |
| Missing | 588 | 6 | 1 |
| **Gestational age at birth (weeks)** |  |  |  |
| <32 | 67,145 (34.08) | 841 (36.76) | 273 (39.80) |
| 32-36 | 37,364 (18.96) | 642 (28.06) | 179 (26.09) |
| 37 + | 92,514 (46.96) | 805 (35.18) | 234 (34.11) |
| Missing | 25,730 | 188 | 44 |
| **Birth weight (g)** |  |  |  |
| < 1500 | 77,953 (35.18) | 830 (33.62) | 259 (35.53) |
| 1500-2499 | 43,018 (19.41) | 855 (34.63) | 221 (30.32) |
| 2500+ | 100,624 (45.41) | 784 (31.75) | 249 (34.16) |
| Missing | 1158 | 7 | 1 |
| **Apgar Score at 5 minutes** |  |  |  |
| <7 | 62,016 (29.53) | 714 (30.57) | 242 (35.12) |
| 07-10 | 148,028 (70.47) | 1,622 (69.43) | 447 (64.88) |
| Missing | 12,709 | 140 | 41 |
| **Mortality** |  |  |  |
| Median [Min, Max] | 9 [0-1824] | 11.5 [0-1774] | 3.5 [0-1,764] |

**CS: Congenital syphilis**
