# Supplementary material for: Mortality in children under 5 years of age with congenital syphilis in Brazil: A nationwide cohort study
Source: PLoS Med. 2023 Apr 7;20(4):e1004209. doi: 10.1371/journal.pmed.1004209 (PMC10081765; doi:10.1371/journal.pmed.1004209)
Supplement: Attachment — Submitted filename: ReviewPLOS.docx [file pmed.1004209.s005.docx]

I would like to congratulate the authors for their preparation of such a well-written and timely manuscript, titled “Mortality in children under 5 years of age with congenital syphilis: a nationwide cohort”

The research question is clearly stated and the relevance to contemporary times cannot be overstated. As the authors astutely pointed out, the majority of long-term sequelae in treated neonates remains unknown. The comparison of infection related variables (both maternal and neonatal) makes this one of the best papers I have read regarding the unknowns of future health for congenital syphilis. Adverse health outcomes have been grossly understudied and through this large population analysis, some clarity about the long-term effects have been demonstrated, controlling for known confounders such as prematurity. The results are interesting, and I am surprised to see that even treated newborns had considerable adverse health outcomes. The strengths and limitations are well described.

I highly recommend this manuscript for publication and look forward to seeing it in print. I am thankful for this opportunity to review such a relevant and well written manuscript.

The methods were clearly defined, and I believe were done using guidelines.

The results were clearly demonstrated and were well described in the manuscript.

I had some questions:

The number of infants dying annually from congenital syphilis is unclear, but estimates suggest over 370 000 perinatal deaths. Do you mean stillbirth and neonatal? Or just neonatal?

In the introduction – you state “globally accepted case definition,” Do you mean for the newborn? If you can explain this briefly.
