# Supplementary material for: Mortality in children under 5 years of age with congenital syphilis in Brazil: A nationwide cohort study
Source: PLoS Med. 2023 Apr 7;20(4):e1004209. doi: 10.1371/journal.pmed.1004209 (PMC10081765; doi:10.1371/journal.pmed.1004209)
Supplement: Attachment — Submitted filename: Point by point ok sub.docx [file pmed.1004209.s006.docx]

**Point by point**

**RE: Mortality in children under 5 years of age with congenital syphilis: a nationwide cohort study**

| **Reviewer #1:** | |
| --- | --- |
| 1. Abstract: I would recommend after the all-cause mortality rate in CS and non-CS groups are given, the overall HR + CI is given (or the crude mortality rate increase given in the third paragraph of the results). | Done. |
| 2. Statistical analyses: "we partially adjusted the model" - I did not follow what partial adjustment meant. | We called the model partially adjusted when we only adjusted for region and year of birth. For the fully adjusted model, we added to the variables from the partially adjusted model + maternal age, education, material status, self-declared race and newborn sex. We made that clearer in methods lines 206-208. We also included this information in the tables. |
| 3. Statistical analyses: for analyses of mortality aged 1-4 years, I am not sure I followed how events in the first year were dealt with. Were they excluded, or was there a time-varying effect of CS included that allowed the mortality rate to change during different time periods? | This analysis was conditional on surviving the first year of life. Therefore, the events in the first year were excluded from the analyses. We made that clearer in methods. Lines 198 and 199. |
| 4. Results "those with a titter higher than 1:64 was more than 9 … times more likely to die than those without CS" technically the hazard ratio is not the same as the risk ratio, although for small event rates it is approximately equal. | We have amended this paragraph:  "During the neonatal period, the hazard ratio among those with a titter higher than 1:64 was 9·35 (95% CI, 7.84-11.15) times higher than among those without a CS registry " |
| 5. Discussion: I agree with the authors's interpretation that linkage error would probably lead to underestimates of association. I wonder if another way in this happens is if CS causes reduction in live birth rate - might this bias the estimates of mortality difference conditional on live birth? | Yes, that could potentially have that effect since gestational syphilis increases the chance of stillbirth. However, in this study, we investigated CS's effects among those born alive. |
| 6. Discussion: I would mention that there is always the chance of unobserved confounders that might cause bias with this study. | We included this limitation in the Discussion. Lines 375 and 378. |
| **Reviewer #2:** | |
| I highly recommend this manuscript for publication and look forward to seeing it in print. I am thankful for this opportunity to review such a relevant and well written manuscript. | We appreciate your time and comments. |
| The number of infants dying annually from congenital syphilis is unclear, but estimates suggest over 370 000 perinatal deaths. Do you mean stillbirth and neonatal? Or just neonatal? | The term perinatal deaths include stillbirths and neonatal deaths. |
| In the Introduction - you state "globally accepted case definition," Do you mean for the newborn? If you can explain this briefly. | Yes, the diagnosis of maternal syphilis is straightforward. However, the diagnosis of congenital syphilis is complex because most infants are asymptomatic, and no reliable laboratory test identifies asymptomatic CS at birth. We included that in the introduction. |
| **Reviewer #3** | |
| 1. During 2014-2017, there was a global shortage of penicillin. This had a major impact on the availability of aqueous, procaine and benzathine penicillin in Brazil for treatment of maternal and congenital syphilis. This needs to be clearly stated and referenced. Below are two references but there are other published data on the impact in Brazil. | We agree that this is an important topic and have included this information in the introduction and discussion lines 100-103 and 360-370. Thank you for pointing this out. |
| 2. Related to item 1, acknowledging the critical impact of shortages of penicillin had on the treatment of maternal and congenital syphilis is an important limitation of the analysis as some of the HR and increased mortality may be associated with the fact that "alternative-non-recommended, non-penicillin" therapies were given to both mothers and infants during the time period of this study. | We agreed with this point, included it as a study limitation, and suggested further research addressing this topic. Lines 360-370. |
| 3. The authors have access to the treatment regimens given to the infants with CS. Where children that received non-recommended treatment for CS more likely to die? I would recommend that a frequency table be added describing the treatment regimens given to CS infants and that a focused analysis of "non-penicillin regimens" and risk of death be considered  4. Should receipt of a non-penicillin treatment in the mother or the infant be included in the multi-variate model for risk of death? | We do have access to the treatment regimen, and we described the frequency in the second paragraph of the results "Of 93 525 live-born children registered with CS, 88 965 had information on live birth treatment. Of those, nearly 95% (84 302/88 965) of the cases were treated, and 56·89% (50 613/ 88 965) received aqueous crystalline penicillin, 12·41% (11 037 /88 965) received procaine penicillin and 7·49% (6 660/ 88 965) received benzathine penicillin. Almost 18% (15 992/ 88 965) received another treatment scheme. More than 71% (369/ 518) of infants who died untreated died within the first 24 hours of life, increasing to 87·06% (451/ 518) by the end of the first week."  However, as we explained in the Discussion, we decided not to include a table estimating the HR by treatment scheme because these analyses would have suffered from selection bias. "Frail live births—i.e. children at a high risk of mortality—will be more likely to receive the complete treatment regime with aqueous crystalline penicillin. Therefore, this treatment will be associated with higher mortality risk and biased interpretation of lower effectiveness".  We also don't know if the "alternative scheme" mentioned in the form is a non-penicillin treatment. Therefore, we included this as a limitation of the paper.  We only have a dichotomous variable (treated or not treated) for maternal treatment, and we used this information. |
| 5. The authors use a global estimate of 370,000 annual from outdated references 9 and 10 in the Introduction and the Author summary second bullet. Would recommend using the 2016 global estimates from reference number 6 Korenromp et al and not including ref 9 and 10. | Thank you. We replaced reference 9 in the author summary. Still, we kept references 9 and 10 in the Introduction because, in that paragraph, we address the number of deaths related to congenital syphilis, and we did not find a more recent reference with this information. |
| 6. This reviewer is aware of a period when an additional criteria for CS was "non-treatment of the male partner of the mother" . If true, over counting of CS cases would have occurred during this period diluting the risk of death from CS. The authors can research and confirm or delete. | Yes, before 2017, the case definition for congenital syphilis included the concomitant treatment of the partner as an epidemiological criterion to be considered in the investigation. We accept that the more sensitive and less specific criterion (relevant since the lack of partner treatment can cause maternal reinfection and lead to congenital syphilis) might have resulted in a sub estimation of fatality risk. |
| 7. What is the definition of "abortion". Is this the same thing as "stillbirth". Abortion may be interpreted as an intentional act to "abort" the pregnancy. | Abortion refers to a termination of pregnancy before 22 weeks, which could be intentional or not, and stillbirth is a foetal loss that occurs after 22 weeks.  To avoid misinterpretation, we replaced the term abortion with the term fetal loss that occurred before 22 weeks of gestation. |
| 8. For classification of child mortality, "under five mortality" overlaps with the other three categories. Be careful how this is used in the paper | Thank you. Yes, there is overlap, we are aware and we’re careful when commenting on it. |
| 9. In the statistical analyses, you state" covariate included in the adjustments were selected a priori based on the literature. Exactly what are the covariates? Please include these. | Thank you. We included this information in lines 206-208. |
| 10. In the Discussion, could bacterial meningitis be neurosyphilis? | It could be, but it could also be caused by another bacteria, and we do not have the relevant clinical data to know. We included this lack of relevant clinical data as a study limitation. |
| 11. In the results and limitations would note that this analysis included "reported" cases. Overcounting as well as undercounting may have occurred | Yes, and we included this in the study limitation lines 376-378. |
| 12. Would add "access to penicillin" treatment in the list of "improvements" in care in the Discussion. | Thank you for this valuable point. We have included it in the list of improvements. |
| Minor edits: 1. Please replace "titters" with "titers" throughout 2. In reference to non treponemal tests replace "titles" with "titers" 3. Please replace "reagent" with "reactive" when referring to a "reactive" non-treponemal test 4. Please define "Brazilian territory" 5. Would rephrase "child biopsy" in procedures 6. Throughout the abstract and text there is use of …f or example About 85% or 84.9%. Please use the same method of data presentation throughout. Also for each percent (%), please provide the numerator and denominator of these calculations. 7. Recommend interpreting the hazard ratios as "risk of death" in the results section | Done. |
| **Reviewer #4:** |  |
| [1] The authors cite in the authors' Introduction that there is an alarming increase in congenital syphilis in several countries. This is true, as there are several publications that report this problem. Therefore, I recommend that authors include these data in the Introduction. Pointing out the countries with these problems is essential to make the relevance of the work clearer, without the reader needing to read its references in order to have this understanding. Of course, it doesn't need to be very detailed (this is in the reference used), but it is important to better qualify the information. | Thank you, we have named the countries with the rising rates of congenital syphilis. Introduction lines 96-97. |
| How did the authors follow all live births from 2011 to 2017? The authors need to explain this part better. | Brazil has mandatory national health registries that allow us to track events throughout the individual's life, including death. Therefore, the follow-up occurred using the record linkage strategy in this birth cohort, including all live births from Brazil. We have now included this information in the manuscript. "The follow-up was designed using the record linkage strategy, by liking birth with death information", and in the summary. |
| Therefore, I recommend authors to better describe the tool used, how it works and if there are already scientific publications that have validated the tool. If there is an article published about the tool, I recommend quoting it, otherwise the authors should describe how the linkage validation tests were carried out on the data used - no matter how good these tools are, they cannot linkage with 100% integrity (this is even mentioned by the authors), similar to models based on relational algebra. What search and similarity algorithms are used in this tool? What is the level of accuracy of these algorithms?. How were exceptions not found handled? How was unrelated data highlighted? What are the limitations of each Information System and how were they treated in the data pre-processing (before linkage)? | Yes, the manuscript published by Barbosa et al. details the record linkage process, including the performance of the CIDACS-RL tool compared with other available tools and the pre-processing phase, which includes data cleaning and standardization. We have now included the information on this paper under the linkage section of the paper.  We also discuss in the limitations of the paper the consequences of linkage error and the use of routine datasets. |
| [2.2] The authors cite three information systems used, which store sensitive patient data in a non-anonymized manner, therefore, the authors must explicitly inform in the methodology that they were authorized by the Ministry of Health to use this information for research purposes, this will protect the article in relation to possible questions about the General Data Protection Law (LGPD) of Brazil. | Thank you. We have included this information: lines 169-170. |
| [2.3] How many records were processed, to later linkage the data? This information does not appear in the methodology. | In the pre-processing phase, we do not exclude any record, even those with missing information. Therefore, all records were submitted to data linkage. |
| [2.4] Notifications of Congenital Syphilis in Brazil are almost not investigated, there is a dissonance between notifications and the investigation of cases for effective confirmation if in fact the child has congenital syphilis. In Brazil today, according to the PCDT, in case of doubt, the notification of congenital syphilis is made, that is, many children may be being notified without actually having congenital syphilis. This subjective criterion included in the most current PCDT and the lack of investigation of cases may be increasing the notification of cases, in addition to the lack of adequate management of cases from prenatal care. How did the authors handle this? In the methodology, they explained which cases were considered, but this issue was not explained. | We used the classification available in the SINAN system. We agree with the referee about the potential misclassification of cases and included this as a limitation of the study. "The diagnosis of congenital syphilis is complex, and the surveillance system might not capture all the cases; therefore, misclassifying exposure status is possible." |
| [3] "We followed 20 057 013 live-born children from birth up to five years", the authors need to better explain how this follow-up was performed. | We have included this information in the methods section lines 130-131. |
| The Discussion presented by the authors is well qualified, but they could go even further. Discuss weaknesses in the reporting system regarding congenital syphilis - SINAN currently appears to be an obsolete system for providing qualified and timely information about the syphilis epidemic in Brazil - The syphilis bulletin is published only once a year, how to make timely decisions looking back? | This is correct, and we agree with the referee's point. |
| In Brazil, syphilis cases are not managed during management until the baby's birth. This is also a reflection of the obsolescence of information systems, the lack of interoperability, and mainly, the lack of integration between the areas of surveillance and health care. This is a point that could also be addressed in the Discussion of the article - the authors should evaluate this possibility. Several articles published, more recently, by Brazilian scientists on syphilis deal with this topic. | We agree and add this point in lines 390-391. |
| In Brazil, the recommendation to create vertical transmission investigation committees may be harming the investigation of cases. It should be noted that WHO and PAHO recommend that cases be investigated, but they do not talk about creating a committee for this. Recent surveys report that such committees do not work for the most part, which impairs the qualification of epidemiological data. | Thank you for your comment. |
| Brazil declared a syphilis epidemic in 2016 and since 2017, several actions have been initiated to respond to congenital syphilis, one of which is the Project "No Syphilis", which was developed jointly with the Ministry of Health and PAHO. One of the major contributions of this technical cooperation project was the inclusion of syphilis in the public health agenda of states and municipalities. Considering that the authors made notes about the context of syphilis in Brazil in the Discussion, it would be important to go deeper and include the actions that are underway in the country and what has already been developed. | We agree and have included a paragraph in the Discussion. “Since 2017, the Brazilian Ministry of Health has implemented actions to respond to the syphilis situation in the country. Several nationwide strategies have been implemented, such as centralized acquisition and distribution of diagnostic and treatment supplies, tools for disseminating strategic information to help decision-making locally, and research development.” |
| **Reviewer #5:** |  |
| The authors acknowledge its limitations, and these limitations do not reduce the importance of the study. My opinion is in favor of accepting the paper for publication in this journal. | Thank you very much |
| **Academic Editor** | |
| 1) It would help to have a figure showing how the total information from the birth registry yielded the numbers studied in detail. | We have included in the supplementary material a flow chart. Figure S1 |
| 2) The cause of death analysis assumes only one ICD code per death, which is a bit odd given misclassification. Could authors provide some information on common combinations by cause? Meningitis in the neonatal period associated with congenital syphilis raises the question of active disease. | We used the underlying cause of death because it is the one which started the succession of morbid events or circumstances that led directly to death. They are used for the production of mortality statistics. However, we searched for congenital syphilis in any line of the death certificate. |
| 3) Some discussion on the completeness of the birth registry and clustering of these deaths, if any, would be useful. | We have added information about completeness in lines 142-143. We do not have the complete address for this dataset, but we evaluated potential clustering at the municipality level, and no clustering was observed. |
| ~~Please include the study setting (Brazil) in your title.~~  ~~In the Abstract Methods and Findings, please include the important covariates adjust for in your analyses.~~  ~~Please add a final sentence to the Abstract Methods and Findings beginning 'The main limitations of this study include…' or similar.~~  The URL in your Data Availability Statement led to a page that never loaded, please check whether this URL is correct (it may have been an issue with the web page itself).  ~~Please include continuous line numbering throughout your manuscript~~.  ~~Please ensure that all numbers in the Abstract match those in the main text.~~  ~~Please define all abbreviations in the Author Summary on first use.~~  ~~Please briefly describe the study design and include headline numbers (sample size and main results) in your Author Summary.~~  ~~Please place citations in square brackets, normal script, and preceding punctuation.~~  ~~Please check throughout for typos (e.g., 'titters' = 'titres').~~  ~~Please confirm whether informed consent was required for this study, and if so, whether this was verbal or written.~~  I could not locate the RECORD checklist, please include this in your Supporting Information. The checklist should be completed using section headings and paragraph numbers (as opposed to page/line numbers).  ~~Did your study have a prospective protocol or analysis plan? Please state this (either way) early in the Methods section.~~ ~~a) If a prospective analysis plan (from your funding proposal, IRB or other ethics committee submission, study protocol, or other planning document written before analyzing the data) was used in designing the study, please include the relevant prospectively written document with your revised manuscript as a Supporting Information file to be published alongside your study, and cite it in the Methods section. A legend for this file should be included at the end of your manuscript. b) If no such document exists, please make sure that the Methods section transparently describes when analyses were planned, and when/why any data-driven changes to analyses took place. c) In either case, changes in the analysis-- including those made in response to peer review comments-- should be identified as such in the Methods section of the paper, with rationale.~~  ~~Please insert tables into the main manuscript, immediately after the paragraph in which they are first cited.~~  ~~Please include Table S1 in the main text and rename as Table 1, as this information is central to the manuscript.~~  ~~Please define CS in the legend of Figure 1.~~  ~~Please include the numerator and denominator when stating percentages in the Results.~~  ~~Please confirm in the Table S2 legend that figures are n (%) unless otherwise stated.~~  ~~Given the observational nature of your study, please avoid causal terms like 'effect' (paragraph one of the Discussion) and refer instead to associations.~~  ~~Please temper claims of primacy (paragraph two, Discussion) by including~~ ~~'to our knowledge', or similar.~~  ~~In your References, please use et al after listing the first six authors.  Please remove and italics formatting in your References.~~ | Done |
|  |  |
